# The patient with recurrent periorbital cellulitis: A sinister NK cell tumor lymphoma

**DOI:** 10.1016/j.ajoc.2025.102335

**Published:** 2025-04-18

**Authors:** L. Dwyer, Q. Qurban, A. Smyth, E. Conlan, H. Fadlelseed, L. Cassidy

**Affiliations:** a Department of Otolaryngology – Royal Victoria Eye and Ear Hospital, Adelaide Road, Dublin, Ireland; b Department of Ophthalmology – Royal Victoria Eye and Ear Hospital, Adelaide Road, Dublin, Ireland; c Department of Pathology, Royal Victoria Eye and Ear Hospital, Adelaide Road, Dublin, Ireland

**Keywords:** Non-hodgkins lymphoma, Pre septal cellulitis, Natural killer cell tumor

## Abstract

**Purpose:**

We describe a rare case of periorbital cellulitis caused by NK cell lymphoma.

**Observations:**

An 18-year-old female presented with left eye periorbital swelling and watering eye for 2 months. She was initially diagnosed with preseptal cellulitis where she had received multiple courses of intravenous antibiotics with no improvement. MRI demonstrated nasolacrimal duct obstruction. The working diagnosis was preseptal cellulitis secondary to dacrocystitis. She was admitted and initiated on intravenous antibiotics. The swelling improved and she was listed for a dacryocystorhinostomy as an outpatient. She subsequently represented with recurrence. A CT orbit and sinus was undertaken which showed severe mucosal disease, and thinning of medial wall of the left orbit. Otolaryngology review was requested, and a functional endoscopic sinus surgery was undertaken with biopsies. Histology demonstrated infiltrative malignancy with Angio centricity and necrosis. The diagnosis was extranodal Natural killer/T cell lymphoma (ENTKL), nasal type.

**Conclusions and importance:**

Primary peri-orbital and intraocular ENTKL has rarely been reported. A high index of suspicion should be maintained in a patient presenting with non-resolving pre septal cellulitis, and a CT sinus should always be considered. Pre- or intraoperative suspicion of malignancy should indicate biopsy, with rapid referral for multidisciplinary team management given the aggressive nature and likely outcome.

## Abbreviations

NK: Natural Killer
EBV: Epstein-Barr virus
NHL: Non-Hodgkins Lymphoma

## 1 Case report

An 18-year-old female was referred by primary care to the ophthalmic emergency department with left eye periorbital swelling and watering eye for 2 months. She was initially diagnosed with preseptal cellulitis where she had received multiple courses of antibiotics with no improvement. She had no history of sinusitis or upper respiratory infection, trauma, infection from a nearby area, or insect bites. There was no history of allergic reactions, trauma, dysthyroid eye disease, chalazion, or hordeolum. The patient had undergone an examination and was diagnosed with an Magnetic resonance imaging (MRI) scan a few weeks earlier in her home country which demonstrated nasolacrimal duct obstruction.

On examination, the eye lid was warm and tender on palpation, with a watery discharge and periorbital swelling. She demonstrated mechanical ptosis, no relative afferent pupillary defect, pupils were equal and reactive to light. Ishihara assessment was 17/17. Vision, globe motility, and intraocular pressures were normal. Diagnostic lacrimal syringing revealed obstruction of the left nasolacrimal duct. There was no associated hemolacrima or swelling. Visual acuity was 20/20 in both eyes with a normal anterior and posterior segment examination. She had no fever or chills and denied diplopia or pain on extraocular eye movement. The working diagnosis was preseptal cellulitis secondary to dacrocystitis. She was admitted and initiated on IV antibiotics flucloxacillin and ceftriaxone. The swelling improved and was listed for a DCR as an outpatient.

**Fig. 1:**
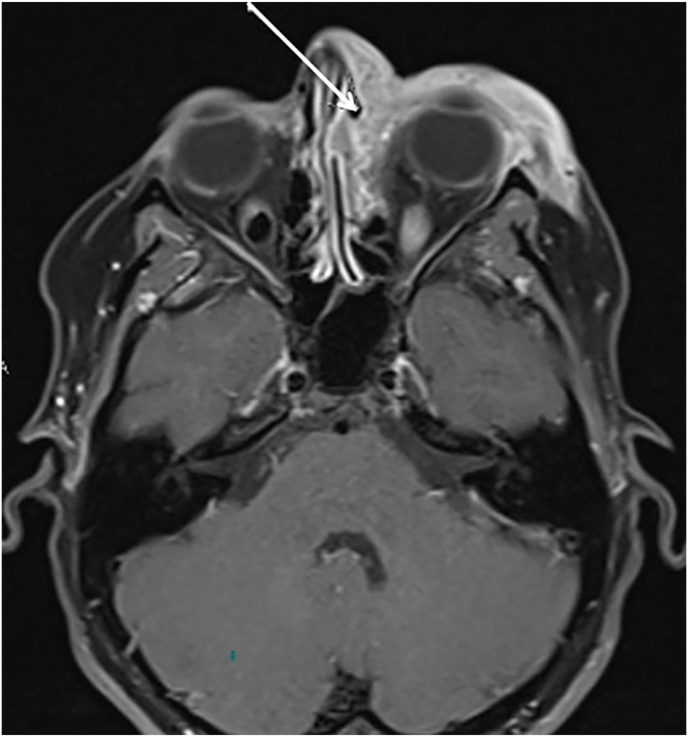

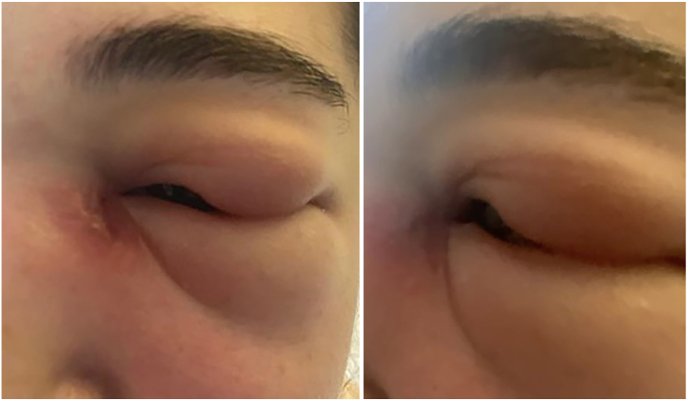
A). MRI orbit demonstrating soft tissue oedema (arrow) surrounding left periorbital region with B). Lateral and frontal clinical photograph suggestive of pre septal cellulitis

She was readmitted 3 weeks later with recurrent periorbital swelling. A repeat MRI was completed which demonstrated left nasolacrimal duct obstruction with secondary abscess of left lacrimal gland (Fig. 1). Furthermore, a computed topography (CT) scan was performed demonstrating a left sinus maxillary echogenic substance. She was recommenced on intravenous antibiotics metronidazole, ceftazidime and vancomycin. All blood cultures and inflammatory profile including c-reactive protein and erythrocyte sedimentation rate were normal. She demonstrated normal full blood count, kidney and liver function. Following the repeated antibiotics, the swelling settled somewhat but the medial canthal swelling persisted. She was subsequently discharged 2 weeks later with planned mucocele surgery in 3 months time.

**Fig. 2:**
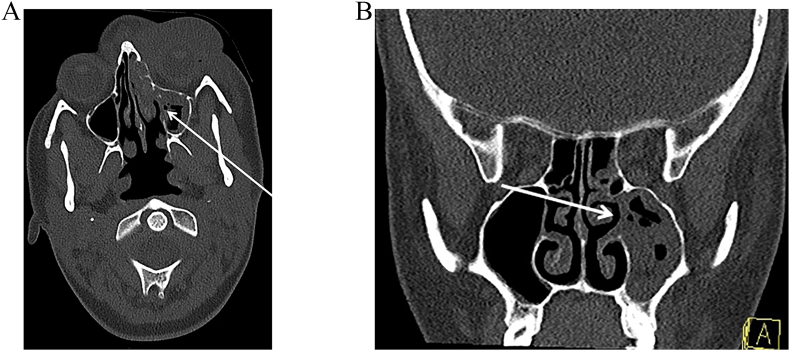
A). Axial section demonstrating, medial wall of left orbit thinning with Stable soft tissue oedema and thickening over the left periorbital region. B). Severe mucosal disease in the left maxillary, left anterior ethmoidal and frontal air cells (arrow). The left osteomeatal complex was occluded, along with the left frontonasal and nasolacrimal duct. The medial wall of the left maxillary sinus was eroded through, with associated mucosal thickening of the middle and superior turbinates. No aggressive osseous lesion was identified. Paranasal sinuses and mastoid air cells were well pneumatised. normal appearances of right orbit and periorbital structures.

Unfortunately, she represented 2 weeks later under our service, with reemergent and persistent periorbital swelling. A CT orbit and sinus (Fig. 2) was undertaken which showed severe mucosal disease in the left maxillary, left anterior ethmoidal and frontal air cells. The left osteomeatal complex was occluded, along with the left frontonasal and nasolacrimal duct. The medial wall of the left maxillary sinus is eroded through, with associated mucosal thickening of the middle and superior turbinates. The medial wall of the left orbit was thinned however it appeared intact. There was stable soft tissue and thickening over the left periorbital region. No aggressive osseous lesion was identified. Paranasal sinuses and mastoid air cells were well pneumatised. normal appearances of right orbit and periorbital structures.

Otolaryngology review was requested, and a functional endoscopic sinus surgery was undertaken with biopsies. This was combined with a septoplasty. It was noted that the sinuses were hypervascular and narrow. Frontal recess clearance with incisional drainage was performed which was filled with thick mucopus. Anterior and posterior ethmoidectomy was undertaken. The lamina papyracea was intact.

On morphology, the biopsy showed dense non-cohesive sheets of atypical lymphocytes with angio-invasive and angio-destructive growth pattern with prominent coagulative necrosis and fibrinoid necrosis (Fig. 3c, Fig. 3a, Fig. 3b). The neoplastic cells demonstratd a spectrum of small lymphocytes to large, atypical lymphocytes with irregular nuclear membrane and pale cytoplasm (Fig. 4). The chromatin was dense in small/medium cells and vesicular in large cells. The neoplastic cells were positive for cluster of differentiation (CD) CD2, cytoplasmic CD3e, CD56, CD8, CD4, CD7 and for both cytotoxic markers; granzyme B and TIA1 gene (Fig. 5, A), B), C) & D)). They are negative for CD5. The histological and immunohistochemical features were consistent with the diagnosis of extranodal NK/T cell lymphoma, nasal type that was Epstein Barr virus positive (EBV). She was subsequently referred for urgent hematological assessment.

**Fig. 3a:**
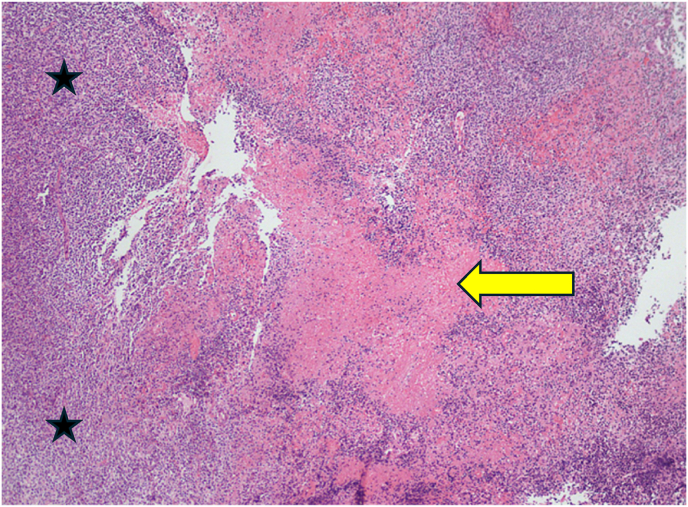
a) Low-power histological view demonstrating diffuse infiltration of lymphoid cells with an extensive coagulative necrosis (Original magnification x4).

**Fig. 3b:**
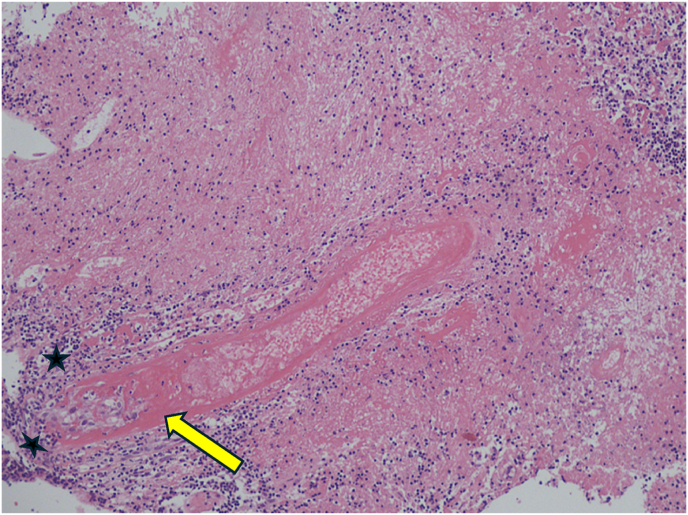
**B**) & C) Showing the neoplastic cells with angiocentric and angio-invasive growth pattern and fibrinoid change in the blood vessel wall (Original magnification x10).

**Fig. 3c:**
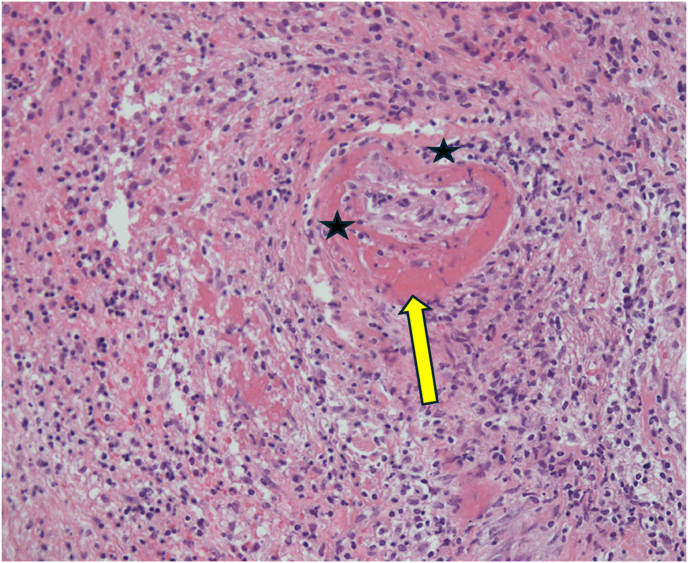
C). (Original magnification x20).

**Fig. 4:**
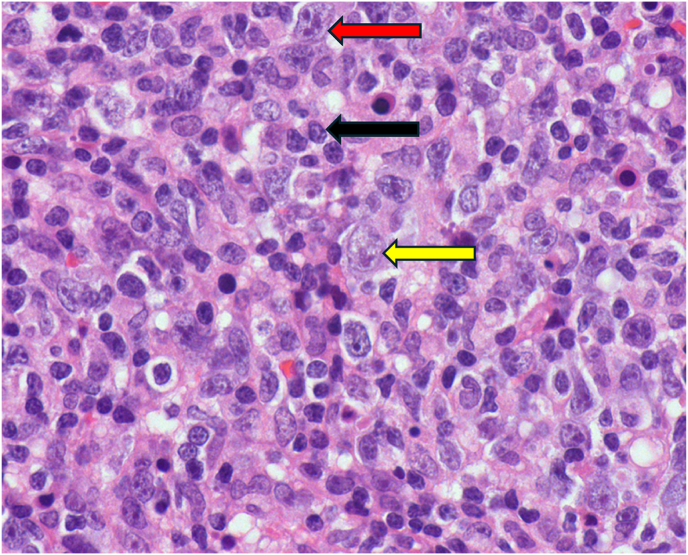
High-power view demonstrating spectrum of diffuse atypical lymphocytes from small to large pleomorphic cells. The neoplastic cells have an irregular nuclear membrane and pale cytoplasm with dense chromatin in small/medium cells and vesicular chromatin in large cells. Some large cells with prominent nucleoli. (Original magnification x60).

**Fig. 5:**
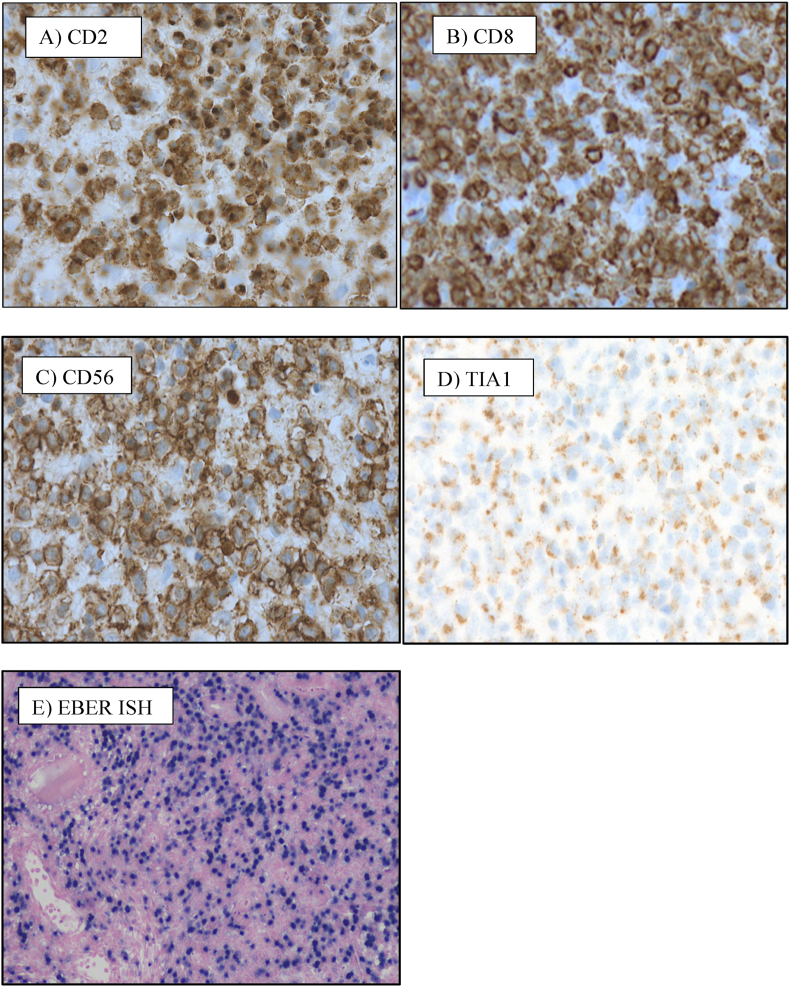
The neoplastic cells demonstrating positive membranous expression for A) CD2, B) CD8, C) CD56 and strong granular cytoplasmic staining for D) TIA1 and nuclear expression for EBER- In situ hybridization.

She was transferred to a tertiary hematological centre for treatment. An chest x-ray demonstrated an incidental right lung nodularity. Positron emission topography CT Showed the left peri orbital soft tissue was consistent with the biopsy-proven lymphoma with an intensely fluorodeoxyglucose (FDG)-avid medial component in the region of the inner canthus/left lateral aspect of the nose and milder FDG uptake within the remainder of the mass. FDG-avid foci within the adjacent left ethmoidal air cells was also suspicious for lymphoma. FDG uptake inferior to the left orbit may represent physiological activity within the inferior rectus muscle but it is slightly asymmetrical compared to the right and therefore a lymphoma infiltration was a possibility. There was asymmetric, moderately FDG-avid right tonsillar focus and mildly FDG avid bilateral subcentimetre upper cervical lymph nodes are indeterminant and may be inflammatory rather than lymphoproliferative in origin although the latter remained a possibility.There was no FDG-avid disease within the thorax, abdomen, pelvis or bones.

Her baseline urea and electrolytes, full blood count and coagulation remained within normal range. Lactate dehydrogenase was raised at 175. She was commenced on intravenous ceftriaxone, vancomycin and metronidazole. Unfortunately given the risk of delayed treatment, fertility preserving treatment was not possible. She was commenced on an urgent non anthracycline based chemotherapeutic regimen.

## 2 Discussion

Preseptal cellulitis is an infection of the skin and soft tissue of the eyelid and surrounding eye.^1^ It affects anterior to the orbital septum and is relatively common in children. Typically, the patient has no pain on eye movements, no visual disturbance, but may be unable to fully open their eyelid.^1^ In many cases, it can result in significant visual and intracranial complications.^1^

Preseptal cellulitis is usually caused by rhinosinusitis. The most common route of spread is from acute ethmoiditis due to the thin perforated bone known as the lamina papyracea representing the only border between the ethmoid sinus and medial orbital border.^2^, ^3^ Despite this, it is important to differentiate preseptal cellulitis from allergic reactions, trauma, orbital tumors, dysthyroid eye disease, chalazion, and hordeolum.

Lymphomas are a hematological malignancy arise from pathological clonal expansion of B lymphocytes or T lymphocytes, and rarely natural killer (NK) cells.^4^ Non Hodgkin lymphoma (NHL) of the head and neck represent 11–33 % cases, and predominantly involve the salivary glands, as well as maxillary and mandibular in 40 % case.^5^^,^^6^ Other less common sites include the paranasal sinuses, waldeyers ring and the orbit.^5^ The majority of lymphomas that arise in the orbit are of low grade, non-Hodgkin B-cell origin.^7^ with the remainder being high grade NK/T cell lymphoma. These non-B-cell type ENKT cell lymphoma account for <1–2 % of all ocular adnexal lymphomas.^8^ The T-cell lymphomas are extremely rare, represent 1–3 % of cases, and usually are a continuance of the “tumor” stage of mycosis fungoides. Systemic NK/T cell lymphoma with secondary involvement is rare.^7^

Patients can present with head and neck lymphadenopathy, facial swelling and numbness, as well as oral ulceration.^9^ Radiological imaging demonstrating bone resorption and lesions with diffuse boundaries should prompt the physician to consider lymphoma. However given the fact that such features can represent other pathologies in the head and neck such as osteomyelitis, NHL's are often accompanied with a late diagnosis and poorer prognosis.^9^ Clinically, ENKT lymphoma is divided into two forms, nasal and non-nasal type. The commonest is nasal type accounting for 80 % of cases. Systemic spread can occur in advanced stage, with involvement of skin, gastrointestinal tract, testis, liver, spleen, or bone marrow. The non nasal type accounts for 20 % of the cases and involves those sites of the advanced stage nasal type like skin, gastrointestinal system, testis, and others.^8^

ENKT cell lymphomas affect males predominantly.^3^, ^10^ They are more common among Asians populations, and are often located in the nasopharyngeal space or nasal cavity.^11^^,^^12^ EBV-positive NK cell malignancies consist of a number of clinical syndromes: Nasal NK cell lymphoma, non nasal/nasal type NK cell lymphoma and aggressive NK cell lymphoma/leukemia. NK cell lymphomas develop mostly in non nodal sites.^13^ The non nasal NK cell lymphomas represent 20 % of cases which involve testis, skin, salivary glands and gastrointestinal tract.^14^ In rare cases, disseminated malignancy may be present with involvement of liver, spleen, lymph nodes and skin. These have involvement of peripheral blood and are known as the aggressive NK cell lymphoma/leukemia.^14^

Morphologically, extranodal NK/T-cell neoplasms characterized by angiocentric and angio-destructive growth pattern with associated prominent coagulative necrosis and fibrinoid necrosis in the blood vessels.^8^ The neoplastic cells have an immunophenotype of T-cell or NK cell lineage.^8^ In most cases the neoplastic cells are positive for CD2, cytoplasmic CD3e, CD56, CD43 and negative for surface CD3, CD4 and CD5. Cytotoxic proteins such as TIA1, granzyme B, and perforin are positive. The differentiation of cell of origin of NK-cell or T-cell lineage is not prognostically significant and it is not routinely required for the diagnosis.^8^The pathogenesis of NK cell lymphomas are strongly linked with EBV which can be detected by In situ hybridization for EBV-encoded small RNA (EBER) (Fig. 5- E).^15^^,^^16^ In such cases, EBV exists in its episomal form and does not integrate into the host genome.^17^ This infection has been shown to occur either before or at the time of lymphoma development, thus in NK cell tumorigenesis. Absence of EBV excludes the diagnosis of NK/T cell lymphoma. The EBV associated NK/T cell lymphoma has an increased risk of macrophages activation and hemophagocytosis.^8^ This leads to a hyper-inflammatory reaction and widespread organ damage.^18^ The most important prognostic factors which are associated with poor prognosis are age over 60 years, advanced-stage systemic involvement and higher levels of circulating EBV-DNA.^8^

## 3 Conclusions

A few cases of periorbital ENKTL have been reported, mostly involving invasion or dissemination of primary nasal lesions; in contrast, primary orbital and intraocular ENKTL has rarely been reported. A high index of suspicion should be maintained in a patient presenting with non-resolving pre septal cellulitis, and a CT sinus should always be considered. Pre- or intraoperative suspicion of malignancy should indicate biopsy, with rapid referral for multidisciplinary team management given the aggressive nature and likely outcome.

## CRediT authorship contribution statement

**L. Dwyer:** Writing – original draft, Visualization, Project administration, Formal analysis, Conceptualization. **Q. Qurban:** Writing – original draft, Project administration, Conceptualization. **A. Smyth:** Writing – original draft. **E. Conlan:** Writing – review & editing. **H. Fadlelseed:** Visualization, Formal analysis. **L. Cassidy:** Supervision.

## Authorship

All authors attest that they meet the current ICMJE criteria for Authorship.

## Funding

No funding or grant support

## Declaration of competing interest

The authors declare that they have no known competing financial interests or personal relationships that could have appeared to influence the work reported in this paper.
